# Altered Rolandic Gamma-Band Activation Associated with Motor Impairment and Ictal Network Desynchronization in Childhood Epilepsy

**DOI:** 10.1371/journal.pone.0054943

**Published:** 2013-01-28

**Authors:** Sam M. Doesburg, George M. Ibrahim, Mary Lou Smith, Rohit Sharma, Amrita Viljoen, Bill Chu, James T. Rutka, O. Carter Snead, Elizabeth W. Pang

**Affiliations:** 1 Department of Diagnostic Imaging, Hospital for Sick Children, Toronto, Ontario, Canada; 2 Neurosciences and Mental Health Program, Hospital for Sick Children Research Institute, Toronto, Ontario, Canada; 3 Department of Medical Imaging, University of Toronto, Toronto, Ontario, Canada; 4 Division of Neurosurgery, Hospital for Sick Children, Toronto, Ontario, Canada; 5 Institute of Medical Science, University of Toronto, Toronto, Ontario, Canada; 6 Division of Neurology, Hospital for Sick Children, Toronto, Ontario, Canada; 7 Department of Psychology, University of Toronto, Toronto, Ontario, Canada; University of Barcelona, Spain

## Abstract

Epilepsy is associated with an abnormal expression of neural oscillations and their synchronization across brain regions. Oscillatory brain activation and synchronization also play an important role in cognition, perception and motor control. Childhood epilepsy is associated with a variety of cognitive and motor deficits, but the relationship between altered functional brain responses in various frequency ranges and functional impairment in these children remains poorly understood. We investigated functional magnetoencephalographic (MEG) responses from motor cortex in multiple functionally relevant frequency bands following median nerve stimulation in twelve children with epilepsy, including four children with motor impairments. We demonstrated that children with motor impairments exhibit an excessive gamma-band response from Rolandic cortex, and that the magnitude of this Rolandic gamma response is negatively associated with motor function. Abnormal responses from motor cortex were also associated with ictal desynchronization of oscillations within Rolandic cortex measured using intracranial EEG (iEEG). These results provide the evidence that ictal disruption of motor networks is associated with an altered functional response from motor cortex, which is in turn associated with motor impairment.

## Introduction

Childhood epilepsy is associated with a host of cognitive and behavioural deficits, including problems with motor function [1]. The neurophysiological basis of these impairments is poorly understood. Neural oscillations have been linked to functional brain activation [2]–[4], and their synchronization across brain regions is understood to reflect communication within distributed brain networks supporting the performance of tasks [5]. Gamma-band oscillations (>30 Hz) are thought to be particularly important for information processing in the central nervous system as they have been related to cortical activation supporting cognition [6], perception, [7], motor control [8] and sensorimotor integration [9]. Altered expression of gamma oscillations and their synchronization among brain regions has also been associated with various neurological and neuropsychiatric conditions [10], [11], including those affecting motor control [5], [12], [13]. Gamma oscillations are related to the maturation of functional networks underlying cognitive development [14], and are altered in children with learning difficulties [15].

Epilepsy is also associated with the abnormal expression of neural oscillations and their synchronization across brain regions [16]–[18]. Excessive high frequency oscillations (HFOs; >80 Hz) are increasingly being accepted as biomarkers of epileptogenic cortex as they appear to be most prominent within epileptogenic brain regions [19]–[21]. As well, epilepsy is associated with atypical oscillatory dynamics in several other frequency ranges, including gamma oscillations below the HFO range [22], [23]. Altered neural oscillations in multiple frequency ranges, including the gamma-band, have been associated with difficulties in cognitive development in pediatric epilepsy [24], and transient psychological alterations are associated with the desynchronization of functional networks in patients with epilepsy [25]. Knowledge is scant, however, regarding how functional brain responses in various frequency ranges are related to functional deficits in specific domains in childhood epilepsy.

Previously, we demonstrated that seizure-induced alterations in oscillatory synchronization involving Rolandic cortex were associated with motor impairment in a group of children with focal cortical dysplasia (FCD) who were undergoing evaluation for surgery for intractable epilepsy [22]. This effect was evident in multiple frequency ranges, but was stronger at higher frequencies including the gamma-band. These findings suggested that ictal disruption of Rolandic networks may impair the motor system’s ability to recruit normal functional responses, thus leading to motor impairment. The present study tested this hypothesis using magnetoencephalographic (MEG) data collected during a median nerve stimulation paradigm [26] which is known to elicit a functional response from Rolandic cortex [27]. We uniquely demonstrate that these functional gamma-band Rolandic responses are atypical in children with abnormal motor function and associated with motor ability in children with epilepsy. We also provide the first evidence that this atypical Rolandic response (MEG) is associated with ictal desynchronization of motor networks (intracranial EEG; iEEG) in children with epilepsy.

## Methods

### Participants

We investigated motor ability and functional responses from Rolandic cortex in a group of children with FCD in whom the relationship between ictal Rolandic desynchronization and motor impairment has previously been established [22]. Children were excluded from this group if median nerve stimulation was performed under general anesthetic, because this is known to alter cortical responses [28]. The resultant cohort was a group of 12 children (mean age = 11.6 years; SD = 4.1 years; 5 females). Subject demographics and clinical characteristics are presented in Table 1. All subjects were scanned at the Hospital for Sick Children as part of the clinical care for their medically-intractable epilepsy. This study was reviewed and approved by the Hospital for Sick Children Research Ethics Board.

**Table 1:** Patient demographics and clinical characteristics.

Subject	Age atMEG	Age at Seizure Onset	Sex	Epileptic Hemisphere	Handedness	Epileptic Focus	MRI Findings	Motor Function
1	14.9	13	Male	Left	Right	Parietal, Rolandic	Cortical Abnormality, Left Precentral Gyrus	Abnormal
2	13.8	6	Female	Right	Left	Rolandic	Cortical Abnormality, Right Precentral Gyrus	Abnormal
3	12.1	2	Female	Right	Right	Frontal	Cortical Abnormality, Right Frontal Parasagital	Abnormal
4	12.4	6	Female	Left	Right	Rolandic	Abnormal Left Paracentral Lobule	Abnormal
5	15.8	12	Male	Right	Right	Frontal, Temporal, Parietal	Subtle FLAIR change in posterior temporal lobe	Normal
6	4.3	3	Male	Right	Right	Occipital, Parietal	Calcarine Thickening	Normal
7	13.5	10	Female	Right	Right	Parietal	Cortical Abnormality, Right Parietal Lobe	Normal
8	4.2	1	Male	Right	Unknown	Frontal, Temporal, Parietal	Cortical Abnormality, Right Parietal Lobe	Normal
9	12.4	3	Female	Left	Right	Rolandic	Cortical Abnormality, Left PeriRolandic Cortex	Normal
10	13	7	Male	Left	Right	Frontal	Normal	Normal
11	7.2	5	Male	Left	Right	Frontal	Cortical Abnormality, Left Frontal Cortex	Normal
12	15.7	10	Male	Left	Left	Rolandic	Normal	Normal

### MEG Data Acquisition and Analysis

**Figure 1:**
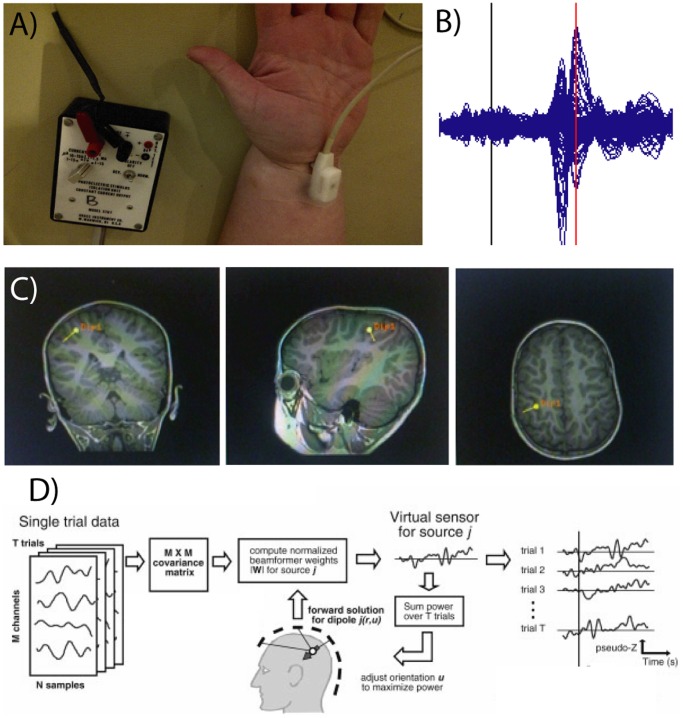
Localization of Rolandic cortex using MEG. Electrical stimulation of the left median nerve (A) produces a high frequency (>30 Hz) response first from right somatosensory cortex, followed by a response from right Rolandic cortex (B); the black line indicates stimulation onset and the red line marks the peak of the functional response from Rolandic cortex. Fitting a single dipole to this second peak reveals a posterior-oriented generator which reliably localizes right Rolandic cortex (C; images in radiological perspective). Using coordinates obtained in this manner, we used beamformer analysis to reconstruct the time series of broadband activity from Rolandic cortex on each trial (D, adapted with permission from Cheyne et al., 2006).

We used data collected during a median nerve stimulation paradigm, which has been described previously [26]. We stimulated the median nerve of each arm (Figure 1A) using a Grass Stimulator (stimulus rate: 5.1 Hz; duration 2.1**µsec, supra-motor threshold). For each subject, 400 trials were recorded with trial length of 200 ms with a 50 ms pre-trigger interval. MEG was recorded at 2500 Hz with a band-pass of 0–833 Hz, and a 60 Hz notch filter to remove line noise and 3^rd^ order gradient noise reduction was employed using a 151 channel MISL Omega system (Port Coquitlam, Canada). Data were epoched and averaged; localization of the generator of the first major neuromagnetic peak following median nerve stimulation using an equivalent current dipole (ECD) method produces an anterior-facing dipole which accurately localizes primary somatosensory cortex, and ECD localization of the second peak produces a posterior-facing dipole which accurately localizes motor cortex (Figure 1B; 1C). This method for localizing motor cortex has been established previously and validated against movement related neuromagnetic cortical responses [27], and is sufficiently reliable that it is used routinely for localization purposes in individual subjects during noninvasive presurgical mapping [26]. We used this ECD method to localize primary motor cortex in both hemispheres by stimulating the contralateral arm, as well as to obtain the latency of the functional response of the motor cortex (peak of the second component).

Broadband time series representing activity from primary motor cortex in each hemisphere, following median nerve stimulation in the contralateral arm, were reconstructed using beamforming [29], which is also an effective means for removing ocular and nonocular artifacts. This method implements a spatial filter which is used to estimate activity at the desired location through a weighted sum of sensor recordings in which activity at all other locations is maximally attenuated without any power change at the reconstructed source location [30]. The entire single-trial epoch length was used to estimate data covariance after band-pass filtering (1–300 Hz). The beamformer weights are then derived from the covariance matrix. Source orientation is then optimized to maximize power for the reconstructed signal for all trials, and broadband single-trial data are then extracted and normalized by estimated noise (3 fT/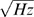), resulting in pseudo-Z values [31]. The process of reconstructing single trial data is further described in Figure 1D, and this has been demonstrated to accurately localize and reconstruct activity from motor cortex [29]. Digital filtering was then used to divide these reconstructed time series into delta (1–4 Hz), theta (5–8 Hz), alpha (8–13 Hz), beta (14–30 Hz), gamma1 (36–44 Hz), gamma2 (45–80 Hz), HFO1 (81–150 Hz) and HFO2 (151–200 Hz) frequency ranges. These frequency ranges were selected in order to facilitate comparison with the iEEG results [22]. We then calculated the analytic signal of the filtered waveforms for each epoch to obtain the instantaneous amplitude at each time point. Instantaneous amplitude values at the time of the second peak, reflecting functional activation of motor cortex, were used to index the amplitude of motor cortex response within each analyzed frequency range.

### Analysis of iEEG Data

The analysis of ictal synchrony involving Rolandic cortex in this group of epilepsy patients has been previously reported [22]. To summarize, subdural electrode grids were surgically implanted and motor cortex was localized in each patient using extra-operative mapping [32]. Localizations of MEG activations elicited during the median nerve stimulation paradigm employed in the present study have previously been shown to have good agreement with locations derived using extra-operative mapping [33]. Ictal periods, identified by an epileptologist, were defined by rhythmic epileptiform activity which evolved over time and which was time-locked with clinical seizures. Interictal periods were two minute segments of activity separated from ictal events by at least one hour. The interictal epochs were selected by experienced electrophysiologists as representative background activity, which in most cases included interictal epileptic discharges. Interictal activity was selected from periods of non-REM sleep according to established methods [26] in order to control for potential state differences in background activity. Ictal and interictal data were filtered into the same frequency ranges used in the present study (see above), the Hilbert transform was used to obtain instantaneous phase values, and phase locking values (PLVs) among electrodes were calculated [34] to index inter-electrode synchrony during the ictal and interictal period. Phase locking was then compared between ictal and interictal (baseline) periods to index ictal desynchronization in each frequency range. Ictal Rolandic desynchronization was defined at the ictal-interictal PLV differences averaged across all electrode pairs within a 3 by 3 group of electrodes centred over the motor hand area [22].

### Assessment of Motor Function

Children underwent neuropsychological assessment and neurological examination during their pre-surgical evaluation. This assessment included testing of motor function using the grooved pegboard test [35] and a finger tapping task [36]. Grip strength in each hand was assessed using a dynamometer. Data from the grooved pegboard task, finger tapping task and grip strength were available for eight patients in this group, as neuropsychological assessments are tailored to the individualized clinical care of each subject. Patients were determined to have abnormal function if they exhibited gross motor weakness on neurological examination or if they performed one standard deviation below average on the grooved pegboard task. This identified four participants in the present study as expressing abnormal motor function. In three of the participants identified in this manner (Subjects 2, 3 and 4), the grooved pegboard task indicated bilateral abnormalities in motor function. In the fourth participant (Subject 1), abnormal motor function was determined by a neurological examination for which laterality information was not reported.

### Statistical Analysis

Data are presented as means with error bars representing standard deviation. Where indicated, binary variables were anlyzed using a two-tailed Fisher’s exact test. The non-parametric randomization test, which employs resampling methods, was used to compare the means of continuous variables. Linear regression was used to determine association between continuous variables. To adjust for confounding variables, a one-way analysis of variance (ANOVA) or analysis of covariance (ANCOVA) was performed for categorical and continuous variables respectively. Outcomes were considered statistically significant at p<0.05. Statistical analysis was performed using SAS Statistical Software 9.3 (Cary, North Carolina).

## Results

### Increased Rolandic Gamma Response in Children with Motor Impairment

**Figure 2:**
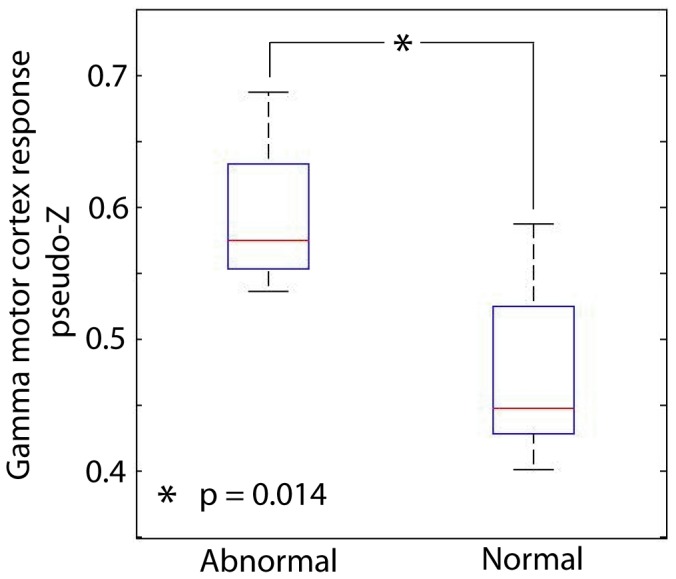
Altered Rolandic MEG response in children with motor impairment. The amplitude of gamma1 (36–44 Hz) response was increased within the epileptogenic hemisphere of children with abnormal motor function.

The magnitude of the functional response from median nerve stimulation was obtained in each analyzed frequency from the signal amplitude (pseudo-Z) at the peak latency of the motor cortex response (second peak associated with dipole with posterior orientation) of each subject. To investigate the relation between functional responses in particular frequency ranges, the amplitudes of the filtered signals were contrasted between children with normal and abnormal motor function, as determined by neuropsychological assessment and neurological examination. Children with abnormal motor function showed greater gamma1 (36–44 Hz) activation in motor cortex within the epileptogenic hemisphere (*p* = 0.014; Figure 2) following median nerve stimulation in the contralateral arm. No statistically significant differences were observed in other analyzed frequency ranges. Since activity in the gamma1 frequency band was most sensitive to differences between normal and abnormal motor function, we focused on this frequency range in subsequent analyses of correlations between neural oscillations in motor cortex and motor function, as well as in the comparison of MEG and iEEG data. The effect of increased Rolandic gamma1 response in children with motor impairments persisted when controlling for sex (*p* = 0.036), age (*p* = 0.020), duration of epilepsy (*p* = 0.034), and whether or not the epileptogenic hemisphere was ipsilateral or contralateral to the dominant hand (*p* = 0.005).

### Altered Rolandic Gamma Response Associated with Motor Ability

**Figure 3:**
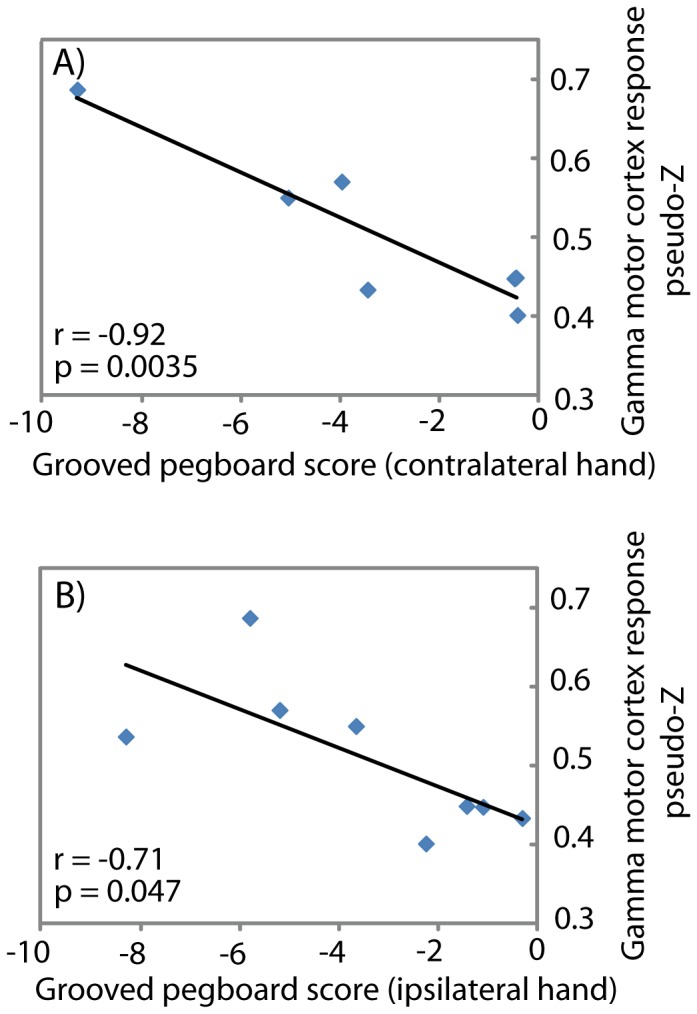
Altered Rolandic MEG response associated with motor ability. Gamma1 (36–44 Hz) response from Rolandic cortex in the epileptogenic cortex was correlated with performance on the grooved pegboard test using both the (A) contralateral, and (B) ipsilateral hand.

To investigate associations between altered gamma1 response and motor ability, we obtained the gamma1 response from each subject at the time of maximal motor cortex response for each subject, using band-pass filtered data from the time series obtained using beamformer source reconstruction from each subject’s functionally mapped motor cortex (see above). Correlation statistics were then employed to assess relations between individual gamma1 responses from motor cortex and motor ability, as indexed by standard neuropsychological assessment. This revealed that the amplitude of gamma1 motor cortex MEG response in the epileptogenic hemisphere was negatively correlated with performance on the grooved pegboard task for both the contralateral (*r* = −0.92; *p* = 0.0035) and ipsilateral (*r* = −0.71; *p* = 0.047) hand (see Figure 3). Trends toward a negative association between the magnitude of the motor cortex gamma response and motor performance measured using the finger tapping task and grip strength were also observed, although these failed to reach statistical significance (Table 2).

**Table 2:** Association of Rolandic gamma response with motor function.

Assessment	Correlation Coefficient (r)
Grooved Pegboard Test, Contralateral Hand	**−0.92** *
Grooved Pegboard Test, Ipsilateral Hand	**−0.71** *
Finger Tapping Test, Contralateral Hand	−0.19
Finger Tapping Test, Ipsilateral Hand	−0.43
Grip Strength, Contralateral Hand	−0.58
Grip Strength, Contralateral Hand	−0.68

* p<0.05.

### Atypical MEG Response Associated with Ictal iEEG Rolandic Desynchronization

**Figure 4:**
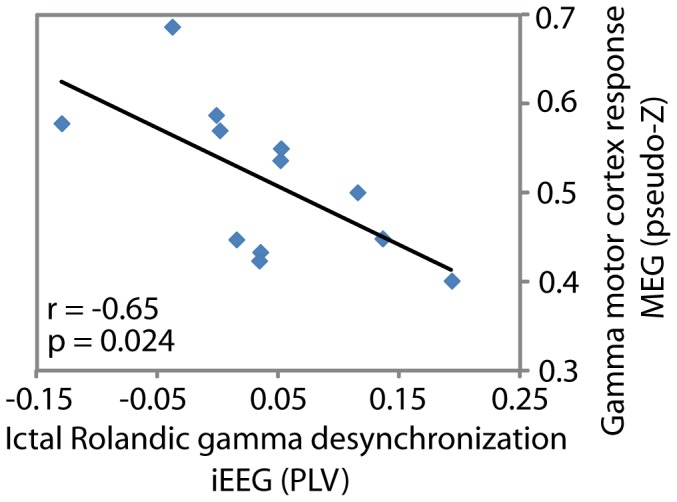
Altered Rolandic MEG response associated with ictal desynchronization. Ictal gamma-band desynchronization of Rolandic cortex, measured using iEEG, was correlated with an atypical MEG gamma-band functional response from Rolandic cortex.

Since the abnormal functional MEG response from motor cortex was within the gamma1 (36–44 Hz) band we focused our examination of the relation between MEG and iEEG data in this frequency range. The analysis of ictal iEEG desynchronization has been previously reported [22]. This analysis revealed that ictal desynchronization was associated with motor impairment in childhood epilepsy. In the present study, we investigated whether ictal desynchronization was associated with an atypical functional response from motor cortex. Indeed, it was found that greater ictal Rolandic desynchronization in the gamma1 frequency range (iEEG) was associated with an increase in gamma1 response amplitude (MEG) from Rolandic cortex (*r* = −0.65; *p* = 0.024). This indicates that greater ictal desynchronization of Rolandic cortex is associated with a more abnormal functional response from motor cortex elicited during median nerve stimulation (Figure 4).

## Discussion

We provide the first evidence that motor deficits in childhood epilepsy are related to an atypical gamma-band response from Rolandic cortex. We also uniquely show that this altered neuromagnetic response is associated with ictal Rolandic desynchronization measured using iEEG. We previously showed that ictal Rolandic desynchronization was associated with motor impairment in children with epilepsy [22], suggesting that seizure induced disruption of network connectivity may cause long-lasting changes which interfere with the motor system’s ability to recruit a normal response. The present study provides evidence that the functional response from Rolandic cortex is indeed atypical in children with epilepsy suffering from motor impairment, and also uniquely demonstrates that this altered Rolandic functional response is associated with ictal Rolandic desynchronization and reduced motor ability. These novel results complement our previous findings and indicate that seizure induced disruption of motor networks leads to abnormal functional responses from the motor system, which in turn leads to impairment of motor function in children with epilepsy. This may provide the basis for a general framework to account for how ictal network dynamics may disrupt the intrinsic organization of eloquent brain networks, inducing long-lasting changes in the system’s ability to recruit a healthy functional response, leading to deficit in the corresponding functional domain.

Our previous analysis of motor network dynamics using iEEG indicated that motor impairment was most strongly associated with differences between ictal and interictal periods, but that ictal activity was much more strongly associated with motor impairment than interictal activity. This, taken together with the fact that MEG responses from sensorimotor cortex are large and robust [26] (see Figure 1B), suggests that observed differences are due to functional responses from Rolandic cortex rather than superimposition of differences in spontaneous interictal activity. Instead, the association of motor impairment with both abnormal functional Rolandic responses and seizure-induced desynchronization of motor networks suggests that encroachment of epileptic patterns of network connectivity on eloquent brain regions negatively impacts the ability of motor systems to engage a normal functional response during motor processing. This view is reinforced by findings that synchronization of gamma oscillations across brain regions can affect regional activation [37] and neural plasticity [38], [39], suggesting that ictal connectivity dynamics may recruit pathological neural plasticity which interferes with functional brain networks.

In the present study, we demonstrate that gamma-band MEG responses from motor cortex are abnormal in children with epilepsy who suffer from motor impairment and show that these atypical responses are associated with individual differences in motor ability in this population. These effects were not attributable to age, sex, duration of epilepsy or whether the dominant hand was ipsilateral or contralateral to the epileptogenic hemisphere. This indicates that the reported findings are not attributable to potentially confounding variables which could impact the amplitude of the Rolandic gamma-band response. The findings of the present study provide new insights into how ictal disruption of functional networks impacts brain activation and function.

The finding that differences between children with normal and abnormal motor function occurred in the gamma1 frequency range is consistent with numerous previous observations which have reliably implicated gamma activity in diverse cognitive, perceptual and motor processes [2], [3], [6], [40]. Moreover, responses from somatosensory and motor cortex following median nerve stimulation are typically mapped on the basis of >30 Hz activity [26] to focus on the dominant frequency content of these functional responses. Gamma activity is understood to reflect the engagement of brain regions supporting task performance across diverse contexts [3], [7], [41], and synchronous gamma oscillations have been proposed as relevant for the coordination of activity across brain regions [42], and likely accounts for the observation that altered brain responses were centred in this frequency range. Previous research has also identified excessive gamma activity in multiple neurological and neuropsychiatric populations [43]–[45], suggesting that this may reflect atypical cortical function across multiple contexts.

The results presented here also conform with a growing body of evidence implicating altered brain oscillations in epilepsy and its associated impairments of function. Excessive high-frequency cortical activity is increasingly being accepted as a biomarker of epileptogenicity [19]–[21], and atypical cortical oscillations in epilepsy also extend into the gamma frequency range shown to be associated with motor impairment in the present study [23]. Pathological brain activity in epilepsy overlaps the functional network activity in frequency space [3], [42] including the gamma-band which has been implicated in the cognitive development [15], and the maturation of functional cortical networks [2]. It is thus not surprising that recent evidence has implicated atypical brain oscillations and their synchronization to problems in cognitive development and psychological alterations in patients with epilepsy [24], [25]. Our results uniquely link epileptic disruptions of oscillatory synchronization, disruption of functional responses from eloquent brain areas, and impairment in epilepsy.

The present study was able to investigate relations among functional MEG responses, ictal iEEG activity and motor function in pediatric epilepsy by taking advantage of clinically collected data. One limitation of this approach is the lack of a control group, which we compensated for by comparing patients with motor impairments with patients without motor impairment. Clinically employed data collection techniques also record very short segments of data which are epoched at collection. This prevented analysis of activity relative to a pre-stimulation baseline. To facilitate comparison with previous results describing relations between ictal desynchronization and motor impairment [22], we studied the same group of children and removed those not suitable for this analysis. Accordingly, the sample size of the present study is small, although many studies with similar sample sizes have been successful in investigating altered brain activity associated with epilepsy. This small sample size also precludes effective analysis of the potential impact of the location of structural abnormalities visible on MRI on altered functional brain responses and their relations with motor function and seizure-induced disruption of motor networks.

### Conclusion

We provide the first evidence that MEG gamma-band responses from motor cortex are associated with motor impairments and ictal desynchronization of Rolandic cortex in children with epilepsy. These results suggest that invasion of eloquent cortical regions by epileptic activity may induce long-lasting changes which negatively affect the brain’s ability to recruit a normal functional response. Such disruption of specific functional brain systems accordingly may lead to impairment in the corresponding domain. These multimodal neurophysiological findings comparing invasive ictal (iEEG) network mapping with interictal noninvasive recordings (MEG) indicate that the long-lasting effects of ictal disruption of functional brain systems can be noninvasively measured using MEG. This strategy promises to provide new means to assess the health of functional brain networks during presurgical planning, to index their function following surgery, and to track the impact of seizures on the function of eloquent cortical areas and networks. The findings of the present study also provide new insights into relations among ictal network dynamics, long-term changes in functional brain activity and developmental difficulties prevalent in pediatric epilepsy.
